# High Prevalence of Epilepsy in an Onchocerciasis-Endemic Area in Mvolo County, South Sudan: A Door-To-Door Survey

**DOI:** 10.3390/pathogens10050599

**Published:** 2021-05-14

**Authors:** Stephen Raimon, Alfred Dusabimana, Gasim Abd-Elfarag, Samuel Okaro, Jane Y. Carter, Charles R. Newton, Makoy Yibi Logora, Robert Colebunders

**Affiliations:** 1 Amref Health Africa, Juba P.O. Box 410, South Sudan; stephenraimon@gmail.com (S.R.); samuel.okaro@amref.org (S.O.); 2 Global Health Institute, University of Antwerp, Kinsbergen Centrum, Doornstraat 331, 2610 Antwerp, Belgium; alfred.dusabimana@gmail.com; 3 Global Child Health Group, Department of Paediatrics and Department of Global Health Academic Medical Center, University of Amsterdam, 1105 BP Amsterdam, The Netherlands; gasim4u83@gmail.com; 4 Amsterdam Institute for Global Health and Development, 1105 BP Amsterdam, The Netherlands; 5 Amref Health Africa Headquarters, Nairobi P.O. Box 30125, Kenya; jane.carter@amref.org; 6 Department of Psychiatry, University of Oxford, Oxford OX3 7JX, UK; charles.newton@psych.ox.ac.uk; 7 Neglected Tropical Diseases Unit, Ministry of Health, Juba P.O. Box 410, South Sudan; morrelogora@yahoo.com

**Keywords:** onchocerciasis, *Onchocerca volvulus*, epilepsy, nodding syndrome, prevalence, ivermectin

## Abstract

In June 2020, a door-to-door household survey was conducted in Mvolo County, an onchocerciasis-endemic area in South Sudan. A total of 2357 households containing 15,699 individuals agreed to participate in the study. Of these, 5046 (32.1%, 95% CI: 31.4–32.9%) had skin itching and 445 (2.8%, 95% CI: 2.6–3.1%) were blind. An epilepsy screening questionnaire identified 813 (5.1%) persons suspected of having epilepsy. Of them, 804 (98.9%) were seen by a medical doctor, and in 798 (98.1%) the diagnosis of epilepsy was confirmed. The overall epilepsy prevalence was 50.8/1000 (95% CI: 47.6–54.4/1000), while the prevalence of nodding syndrome was 22.4/1000 (95% CI: 20.1–24.9/1000). Younger age, being male, skin itching, blindness, and living in a neighbourhood or village close to the Naam River were risk factors for epilepsy. The annual incidence of epilepsy was 82.8/100,000 (95% CI: 44.1–141.6/100,000). Among children 7–9 years old without epilepsy, 34% were Ov16 seropositive, suggesting high ongoing *Onchocerca volvulus* transmission, but only 41.9% of them took ivermectin during the last mass distribution. In conclusion, a high prevalence and incidence of epilepsy was observed in Mvolo, South Sudan. Strengthening of the onchocerciasis elimination programme is urgently needed in order to prevent epilepsy in this region.

## 1. Introduction

A high prevalence of epilepsy, including nodding syndrome (NS), has been observed in onchocerciasis-endemic areas in South Sudan [1,2,3,4]. NS was first investigated by a team from the World Health Organization in 2011 in Mundri (Lui and Amadi) in Western Equatoria State [1]. In 2013, the epilepsy prevalence in Mvolo County in Western Equatoria was estimated by the South Sudan Relief and Rehabilitation Commission to be 8.4% (4025/48,100) (Anthony Amba, personal communication). This high prevalence was confirmed during a rapid assessment of the epilepsy prevalence in Mvolo in Western Equatoria. In a survey of 22 households, 28 (16.7%) of 168 children were found to have epilepsy, and in 13 (59%) of the households there was at least one child with epilepsy [3].

In 1948, DJ Lewis, a medical entomologist, described Mvolo as a place with extremely intense *Simulium* spp. biting and high *Onchocerca volvulus* infection prevalence in the flies (in up to 10% of flies L3 larvae were identified in the heads) [5]. He described Mvolo as having only a police post with very few people residing in the area. Today Mvolo is a town surrounded by an agricultural area, where it is hypothesized that despite the risk for onchocerciasis, many people have settled because of the fertile land and the ample supply of fish from the Naam River.

Based on data obtained from cross-sectional epilepsy surveys performed before, during, and after the implementation of onchocerciasis elimination programmes, it was suggested that it is possible to stop an NS epidemic and decrease the incidence of epilepsy in onchocerciasis-endemic regions [6,7,8]. However, because the pre- and post-intervention surveys used different methodologies, different epilepsy definitions, and were carried out in different study areas by different research teams, the interpretation of these results is difficult. Therefore, we initiated a prospective study in three onchocerciasis-endemic areas in South Sudan where a high prevalence of epilepsy was reported: Maridi, Mundri, and Mvolo Counties [4]. The aim of this study is to compare the effects of bi-annual community-directed treatment with ivermectin (CDTI)—with and without community-based vector control—with annual CDTI on the incidence of epilepsy, including NS [9]. As part of this initiative, baseline epilepsy surveys were conducted at the three study sites. The results of the survey in Maridi County were published in 2019 [4]. In this paper we present the results of the survey in Mvolo County, located in Western Equatoria State, South Sudan.

## 2. Methodology

### 2.1. Study Setting

**Figure 1:**
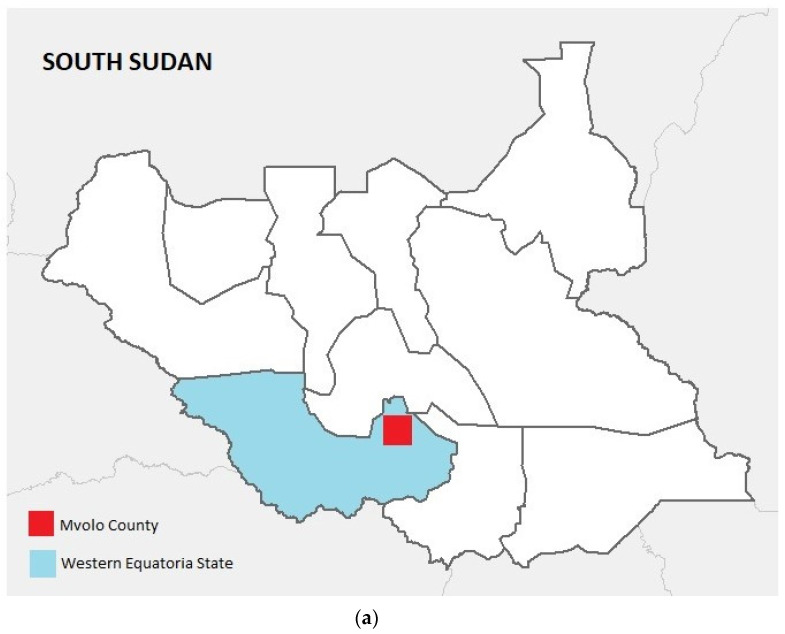

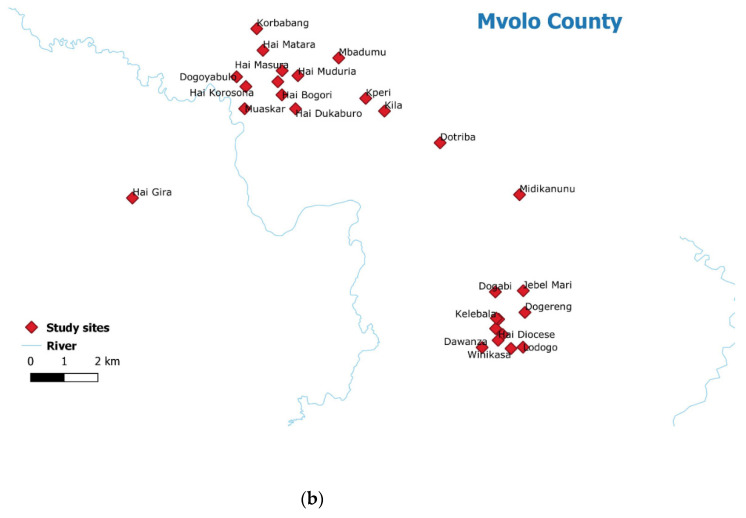
(**a**) Map showing South Sudan with Western Equatoria State and Mvolo County highlighted. (**b**) Map showing the location of Mvolo County, and the neighbourhoods and villages visited during the house-to-house survey.

The study was conducted in villages in the Mvolo (N6.060121, E29.952274) area, located close to the fast flowing Naam River, which is infested with blackflies (Figure 1a,b).

**Figure 2:**
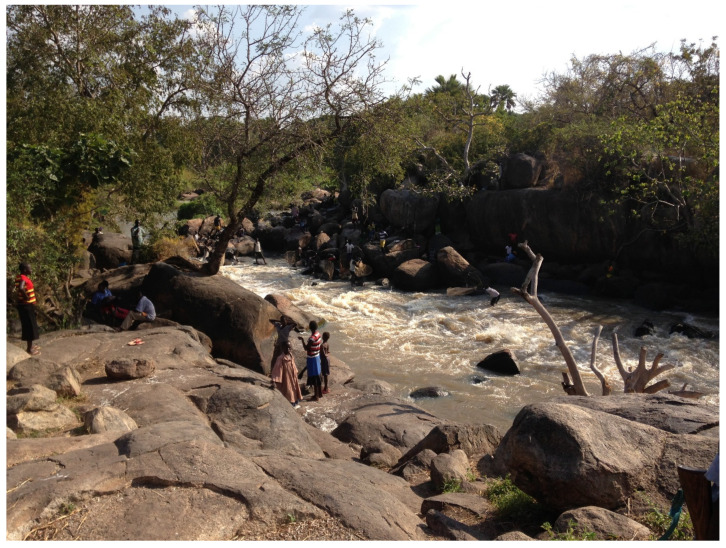
Naam River in Mvolo, a crowded area during the fishing season.

This river becomes very crowded during the fishing season, since it is a focal point for communities where men fish, children swim, women wash clothes, and cows graze along the riverbanks (Figure 2).

The projected population of Mvolo is estimated at 67,864. The Jur are the dominant ethnic group in Mvolo; other ethnic groups include the Nyamusa and the Morokodo. Jur is the dominant language in Mvolo, and the common language of communication is local Arabic.

Farming and fishing are the main economic and livelihood activities; the main crops grown in Mvolo are sorghum, simsim (sesame), groundnuts, vegetables, maize, and millet. Goats, chicken, and cattle are the main livestock in Mvolo; no pigs are kept in the area for cultural reasons.

The distribution of annual doses of ivermectin to residents of Mvolo started in 1996, but treatment was interrupted several times during periods of conflict (S Komyangi, personal communication).

### 2.2. Study Design

A door-to-door survey was conducted in different neighbourhoods and villages in the Mvolo area. Two steps were used to identify people with epilepsy (PWE). In the first phase, households were visited by a trained research team consisting of 20 locally recruited research assistants; each household was visited by one research assistant. After obtaining informed consent, family members were interviewed using a validated questionnaire translated into the local language [10]. In the second phase, persons suspected of having epilepsy by the research assistants were referred to be interviewed and examined by a medical doctor, who either confirmed the diagnosis of epilepsy or suggested an alternative diagnosis. The 20 research assistants were selected by the Mvolo County Health Department, and the 7 medical doctors were selected by the academic affairs office of the College of Medicine at the University of Juba. Research assistants were chosen from among the county’s community drug distributors with at least secondary-school-level education, and were trained in the use of the screening questionnaire for suspected epilepsy during a one-day training workshop. The seven medical doctors were trained in how to confirm the diagnosis of epilepsy. Both the research assistants and medical doctors pilot tested the data collection tools prior to the actual data collection. All of the training was organized by a medical doctor (SR). Moreover, during the home visits, SR supervised the research assistants and the medical doctors, and also interviewed and examined selected persons with suspected epilepsy.

#### 2.2.1. Definitions

A case of epilepsy was defined based on the International League against Epilepsy as being an individual with at least two unprovoked seizures with a minimum of 24 h separating the two episodes [11]. Nodding seizures were defined as the head dropping forward repeatedly in a person during a brief period of reduced consciousness. Onchocerciasis-associated epilepsy (OAE) was defined as a person meeting all of the following six criteria: (1) a history of at least 2 unprovoked epileptic seizures at least 24 h apart; (2) living for at least 3 years in an onchocerciasis-endemic region; (3) living in a village with high epilepsy prevalence and with families with more than one child with epilepsy; (4) no other obvious cause of epilepsy; (5) onset of epilepsy between the ages of 3 and 18 years; (6) normal psychomotor development before the onset of epilepsy.

As potential “obvious causes of epilepsy” we considered a history of severe malaria, encephalitis or meningitis, and head injury with loss of consciousness in the five years preceding the onset of epileptic seizures. Nakalanga features were defined as an association of growth retardation without obvious cause, delay or absence of external signs of secondary sexual development, intellectual disability, epilepsy, and often facial, thoracic, and spinal abnormalities [9]. A person was considered blind if he/she was unable to recognize the five fingers of a hand. A “permanent household” was defined as a family who had lived in the village for at least 20 years. An “immigrant household” referred to a family who had lived in the village for less than 20 years.

#### 2.2.2. Data Collection and Management

Screening of households by research assistants to identify persons suspected of having epilepsy was performed using a paper-based questionnaire (Supplementary Material S1). This questionnaire included five epilepsy screening questions to be addressed to each family member, adapted from a questionnaire that was previously validated in Mauritania [10]. If the answer to one of the five epilepsy screening questions was yes, then this person was considered to be suspected of having epilepsy, and was seen by a clinician. Each household member was also asked whether they had taken ivermectin during the previous distribution. The questionnaire also contained questions about duration of residence, ethnicity, main income generating activity of the family, exposure to cattle or pigs, and whether family members had recently developed epilepsy. For the confirmation of epilepsy, medical doctors used a paper-based questionnaire with unique codes assigned to each suspected case (Supplementary Material S2). Clinicians assessed the type of epilepsy, potential triggers of seizures, potential obvious causes of epilepsy, epilepsy related co-morbidities (cognitive impairment, behavioural problems, burn scars, etc.), onchocerciasis-related clinical signs (skin lesions, vision problems), anti-seizure medication received, and ivermectin intake in the past. The distance of the study villages to the Naam River in Mvolo was estimated using the villages’ GPS coordinates and the point coordinates of the river.

#### 2.2.3. Onchocerciasis Antibody Testing

A total of 150 children aged between 3–9 years, randomly chosen among children living in the Hai Korosona, Hai Matara, Dogoyabulo, or Muaskar neighbourhoods of Mvolo centre, were tested for onchocerciasis antibodies using the Ov16 rapid test (Standard diagnostics, Inc., Yongin-si, Korea) as an indicator of the degree of recent *O. volvulus* transmission. Blood was obtained via finger prick and the test was performed according to the manufacturer’s instructions. Among these Ov16-tested children, we investigated ivermectin use in 2019 for those aged 5–9 years.

### 2.3. Data Analysis

Continuous variables were summarized using the median and interquartile ranges, and frequencies and percentages were used for categorical variables. The prevalence of epilepsy was calculated by dividing the number of clinically confirmed epilepsy cases by the total number of individuals screened. The overall epilepsy incidence in the general population was calculated by dividing the number of new cases of epilepsy in the previous 12 months by the total number of the study population. Ivermectin coverage was defined as the percentage of the population that reported taking ivermectin in 2019. A multivariable logistic regression using the generalized estimating equations (GEE), adjusting for the similarity between the participants from the same family and families from the same village, with logit link function, was used to assess the potential risk factors of epilepsy. All two-way interactions between the predictors were considered in the model, and a likelihood ratio test was used to identify potential interactions.

## 3. Results

In total, 2356 households comprising 15,699 individuals agreed to participate in the survey. The median number of household members was 4 (IQR: 2–6); 2035 (88%) families belonged to the Jur ethnic group. Farming was the main income-generating activity for 2292 (97.3%) families; 374 (15.9%) families reported fishing and 382 (16.2%) reported cattle keeping; 248 (10.5%) considered themselves employees and 177 (7.5%) traders; 1985 (92.0%) families had lived for more than 10 years in the village, while 371 (15.8%) were immigrant families (median (IQR) period of residence 9.0 (4.0–13.0) years).

### 3.1. Prevalence of Potential Onchocerciasis Associated Co-Morbidities

A total of 15,699 individuals from 2357 households agreed to participate in the survey. Of these, 5065 (32.1%, 95% CI: 31.4–32.9%) had skin itching and 445 (2.8%, 95% CI: 2.6–3.1%) were blind (Table 1).

The highest numbers of PWE were observed in Hai Bogori (10.4%) and Hai Korosona (8.8%)—two neighbourhoods in the central part of Mvolo, very close to the Naam River (Table 1).

**Figure 3:**
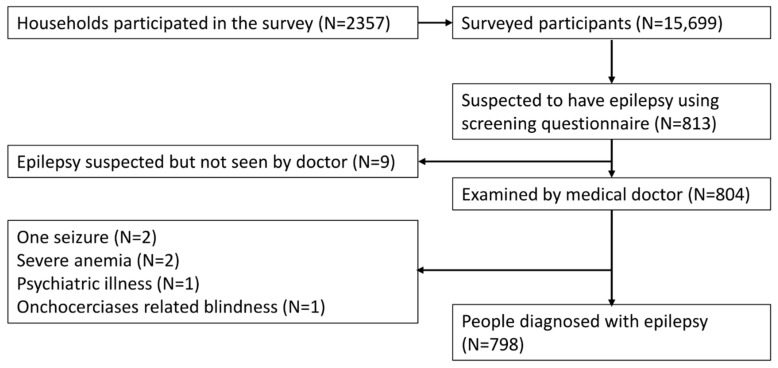
Study participants, from household epilepsy screening to epilepsy diagnosis by the medical doctor.

An epilepsy screening questionnaire identified 813 (5.2%) persons suspected of having epilepsy. Nine individuals suspected of having epilepsy were not seen by a clinician (Figure 3).

### 3.2. Prevalence of Epilepsy

In 798 (98.1%) persons suspected of having epilepsy, the diagnosis of epilepsy was confirmed by the clinician. Of the six persons in whom the diagnosis of epilepsy was not confirmed, two had a history of only one seizure, one had a psychiatric problem, two presented with severe anaemia, and one had onchocerciasis-related blindness without epilepsy. The overall epilepsy prevalence was 50.8/1000 (95% CI: 47.6–54.4), while that of nodding syndrome was 22.4/1000 (95% CI: 20.1–24.7) (Table 1). Epilepsy prevalence was highest in the 16−20- and 21−25-year-old age groups, at 112.3/1000 (95% CI: 100.8–125.8) and 140.7/1000 (95% CI: 122.5–161.1), respectively (Table 2).

### 3.3. Incidence of Epilepsy

Thirteen PWE developed their first seizures in the twelve months preceding the household survey (annual incidence 82.8/100,000) (95% CI: 44.1–141.6/100,000).

### 3.4. Characteristics of Epilepsy

Of the 798 PWE, 378 (47.4%) were female (Table 3). The median age (IQR) of PWE was 20 (17.0−25.0) years. Of the 709 PWE for whom the age of onset of their first seizure was known, the median (IQR) age of onset was 9.0 (6.0−13.0) years. The median (IQR) age of onset of the first nodding seizure was 7.0 (5.0−10.0) years. Thirteen (1.6%) PWE developed their first seizures during the previous year; 729 (91.8%) had at least one seizure in the previous 12 months. The most frequent seizure type was generalized convulsive seizures, reported in 641 PWE (80.3%); 352 (44.1%) were classified as persons with nodding seizures, 82 (10.3%) with only nodding seizures, and 270 (33.8%) had a history of nodding seizures and other types of seizures. In 666 (83.7%) there was no specific trigger that caused the seizures, but in 134 (16.3%) seizures were triggered by the sight of food.

One hundred and sixty-six (28.4%) PWE presented with cognitive impairment, and in fifty-one (8.7%) there were behavioural problems. Papular nodular pruritic lesions were observed in 63 (7.9%) PWE, and burn lesions in 115 (14.4%); 302 (37.8%) reported skin itching. Six hundred and forty-four (80.7%) PWE were taking anti-seizure medications, mainly carbamazepine (545: 68.3%). Six hundred and sixty-one (82.8%) had previously taken ivermectin, and six hundred and forty-five (80.8%) had taken ivermectin in the year preceding the survey (Table 3).

**Figure 4:**
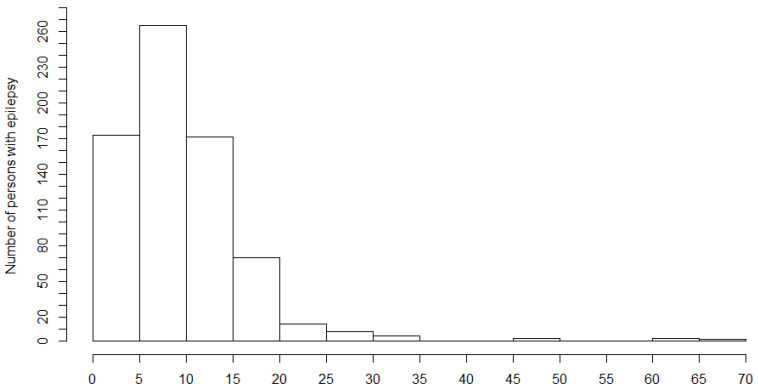
Age of onset of the first seizure among persons with epilepsy.

The first seizures appeared most frequently between 5 and 10 years of age (Figure 4).

A large number 556 (78.4%) of the 709 PWE for whom information about the criteria of OAE was available met these criteria. In 483 (60.8%) of PWE there was a history of epilepsy in the family, and in 366 (75.6%) of them this was a sibling. Nakalanga features were observed in 51 PWE (persons who were more than 20 years old and “looked like a child”). Of these, 30 (58.8%) were male. Two (3.9%) had facial abnormalities and ten had (19.6%) thoracic or spinal abnormalities. In 7 (13.7%) of the 21 women, at 20 years old and above, breasts were not developed.

### 3.5. Risk Factors for Epilepsy

**Figure 5:**
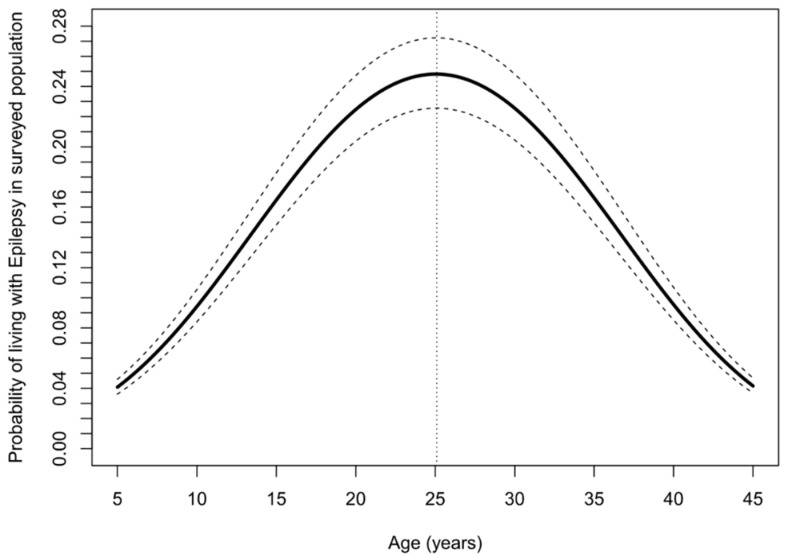
Adjusted probability of living with epilepsy as a function of age. The solid line represents point estimates, while the dashed lines represent 95% confidence bands.

Younger age, male sex, skin itching, blindness, and residing in a neighbourhood or village close to the Naam River were important risk factors for high prevalence of epilepsy (Table 4). The probability of living with epilepsy increased with increasing age up to 25 years, but decreased above this age (Figure 5).

Of the 798 PWE, 645 (80.8%) had taken ivermectin in 2019, compared to 9859 (71.5%) of the 13,780 ivermectin-eligible study population (>4 years old) (*p*-value < 0.001).

The overall Ov16 seropositivity among the children without epilepsy below 10 years of age was 27.3%, but 34% among children 7–9 years old. (Table 5).

Ivermectin intake among children who were eligible for ivermectin treatment during the last mass distribution was 41.9% among the 7–9 year old children, and even lower in younger children.

## 4. Discussion

Our study confirms the high prevalence and incidence of epilepsy in the Mvolo area in South Sudan. This prevalence (50.8/1000) is higher than the 44/1000 epilepsy prevalence reported in Maridi [12]. A large proportion of PWE (78.4%) met the OAE criteria. Only 44.1% had a history of nodding seizures. This is similar to the 45.5% reported in Maridi [12]. A large percentage of the study population reported itching (32.1%), and 2.8% were blind. These findings suggest a very high level of past and ongoing *O. volvulus* transmission. This high level of transmission could be explained by the low past coverage of community-directed treatment with ivermectin in the general population. A higher number of PWE had taken ivermectin in 2019 (80.8%) compared to the general population (71.5%) (*p* < 0.001). A potential explanation for this could be the recent epilepsy onchocerciasis awareness campaigns, which were launched to increase ivermectin coverage of eligible individuals. Despite the WHO recommendation that ivermectin treatment should not be given to severely ill individuals, today it is universally accepted that epilepsy should not be considered a contraindication for ivermectin use. On the contrary, there is evidence that ivermectin, in particular when given twice a year, may decrease the frequency of seizures in *O. volvulus*-infected PWE [13].

Younger age, male sex, family income from an activity other than farming, skin itching, blindness, and residing in a neighbourhood or village close to the Naam River were risk factors for a high prevalence of epilepsy. Cattle keeping was not associated with a lower prevalence of epilepsy. It has been suggested that the presence of cattle may protect cattle herders from developing onchocerciasis-associated disease because blackflies may bite the cattle, and therefore bite humans less often, as well as because of cross-protecting *Onchocerca ochengi* antibodies [6,14]. It is however unknown whether *O. ochengi* infections are present in cattle in the Mvolo area.

Residing in a village close to the Naam River was associated with higher prevalence of epilepsy. The Naam is a rapidly flowing river and a blackfly breeding site. Therefore, persons living close to this river are often exposed to the bites of *O. volvulus*-infected blackflies. Many studies have reported a higher prevalence of epilepsy in villages closer to the river [4,15].

We did not perform Ov16 testing, nor skin snip testing, to detect *O. volvulus* microfilariae in persons meeting the criteria of OAE, because we know from other studies that in the majority of them there would be parasitological evidence of an *O. volvulus* infection [1,16]. However, we performed Ov16 testing in children aged between 3–9 years. The high prevalence of *O. volvulus* antibodies in children, already at a very young age, indicates a high level of ongoing *O. volvulus* transmission, and explains the high onchocerciasis disease burden in the Mvolo area. Particularly worrisome is the low ivermectin intake in of children age below 10 years. Such children are at risk of developing a high *O. volvulus* microfilarial load, which puts them at risk of developing OAE [17,18].

In recent years, the association between onchocerciasis and epilepsy has been clearly established in many epidemiological studies [19]. Moreover, these studies showed the usefulness of the OAE case definition for identifying hotspots of high ongoing *O. volvulus* transmission [20]. However, the pathophysiological mechanisms of OAE still need to be elucidated.

The strength of our study is that all households were visited by a research team that included a clinician who was able to confirm or reject the diagnosis of epilepsy at the home of the PWE. However, several limitations of the study need to be mentioned. We did not systematically assess the presence of onchocerciasis nodules during clinical examination, and no *O. volvulus*-specific tests were performed on persons with epilepsy. Moreover, neither laboratory studies nor imaging investigations were performed to identify the causes of epilepsy.

## 5. Conclusions

This study confirms the high prevalence of epilepsy, including nodding syndrome, in an onchocerciasis-endemic area in which the onchocerciasis elimination programme was working sub-optimally. Strengthening the local onchocerciasis elimination programme is urgently needed in order to prevent children in Mvolo from developing OAE.

## Figures and Tables

**Table 1:** Prevalence of potential onchocerciasis co-morbidities including confirmed epilepsy by study neighbourhood/village.

Village	Distance to the Naam River (in km)	Participants in Survey	Persons with Epilepsy, *n* (%)	Itching, *n* (%)	Blind, *n* (%)
Dogoyabulo *	0.7	377	20 (5.3)	111 (29.4)	6 (1.6)
Dokaburo *	2.3	93	3 (3.2)	17 (18.3)	9 (9.7)
Hai Bogori *	1.9	677	70 (10.3)	210 (31.0)	32 (4.6)
Hai Dileb *	1.8	194	6 (3.1)	26 (13.4)	19 (9.8)
Hai-Gira *	4.3	1181	93 (7.9)	467 (39.5)	38 (3.2)
Hai Korosona *	0.9	674	59 (8.8)	172 (25.5)	21 (3.1)
Hai Matara *	1.9	1205	62 (5.1)	341 (28.3)	23 (1.9)
Hai Masura *	2.0	230	16 (7.0)	61 (26.5)	6 (2.6)
Mbadumu *	3.7	551	33 (6.0)	123 (22.3)	26 (4.7)
Muaskar *	0.9	17	2 (11.8)	4 (23.5)	0 (0.0)
Muduria *	0.9	484	24 (5.0)	105 (21.7)	9 (1.9)
Hai Diocese	35.6	551	30 (5.4)	175 (31.8)	19 (3.4)
Hai Malakia	33.7	363	20 (5.5)	107 (29.5)	5 (1.4)
Hai Zira	34.4	572	26 (4.5)	145 (25.3)	21 (3.7)
Dawanza	35.4	89	3 (3.4)	36 (40.4)	3 (3.4)
Dogabi	31.2	217	8 (3.7)	54 (24.9)	5 (2.3)
Dogereng	34.7	689	34 (4.9)	279 (40.5)	18 (2.6)
Dokati Ngobo	6.1	379	11 (2.9)	118 (31.1)	8 (2.1)
Dolo	24.3	216	3 (1.4)	20 (9.3)	2 (0.9)
Domeri	13.4	565	48 (8.5)	264 (46.7)	13 (2.3)
Dotriba	6.7	866	21 (2.4)	435 (50.2)	28 (3.2)
Jebel Mira	32.0	107	8 (7.5)	39 (36.4)	0 (0.0)
Kelibala	33.5	347	11 (3.2)	84 (24.2)	3 (0.9)
Korbabang	30.0	712	15 (2.1)	244 (34.3)	7 (1.0)
Kperi	4.4	938	44 (4.7)	159 (17.0)	39 (4.2)
Kulu	5.0	621	25 (4.0)	138 (22.2)	33 (5.3)
Lamu	26.0	364	5 (1.4)	124 (34.1)	7 (1.9)
Lodogo	37.6	80	3 (3.8)	24 (30.0)	4 (5.0)
Medikanunu	9.4	321	15 (4.7)	135 (42.1)	5 (1.6)
Minikolome	7.7	1026	39 (3.8)	332 (32.4)	17 (1.7)
Tiboro	22.1	281	22 (7.8)	118 (42.0)	7 (2.5)
Winikasa	37.0	261	5 (1.9)	90 (34.5)	11 (4.2)
Winikelo	5.3	107	6 (5.6)	67 (62.6)	0 (0.0)
Yeri Centre	35.4	344	8 (2.3)	222 (64.5)	1 (0.3)
Overall		15,699	798 (5.1)	5046 (32.1)	445 (2.8)

* Neighborhoods of Mvolo center.

**Table 2:** Prevalence of nodding syndrome and other forms of epilepsy by sex and age group.

	Total Number of Participants	Persons with Epilepsy, *n* ‰ (95%CI)	Nodding Syndrome, *n* ‰ (95%CI)
Sex			
Female	7847	378 (48.2, 43.7–53.1)	168 (21.9, 18.8–25.5)
Male	7845	419 (53.4, 49.7–58.6)	184 (23.5, 20.3–27.0)
Age groups in years			
0−4	1919	6 (3.1, 1.4–6.8)	3 (1.6, 0.5–4.6)
5−10	2928	31 (10.6, 7.5–15.0)	20 (6.8, 4.4–10.5)
11−15	2105	122 (58.0, 48.8–68.8)	67 (31.8, 25.1–40.2)
16−20	2459	277 (112.3, 100.8–125.8)	138 (56.1, 47.7–65.9)
21−25	1244	175 (140.7, 122.5–161.1)	73 (58.7, 46.9–73.2)
26−30	1447	109 (75.3, 62.8–90.1)	34 (23.5, 16.9–32.7)
31−35	754	35 (46.4, 33.6–63.9)	10 (13.3, 7.2–24.2)
36−40	1079	24 (22.1, 15.0–32.9)	4 (3.7, 1.4–9.5)
41−45	492	6 (12.2, 5.6–16.3)	1 (2.0, 0.4–11.4)
>45	1272	13 (10.2, 6.0–17.7)	2 (1.6, 0.4–5.7)
Overall	15,699	798 (50.8, 47.6–54.4)	352 (22.4, 20.1–24.7)

**Table 3:** Characteristics of the 798 persons with epilepsy.

**Characteristics**	
Female, *n* (%)	378 (47.4)
Age (years), median (IQR)	20 (17.0–25.0)
Born in the village, *n* (%)	731 (91.6)
Period (years) of residing in the survey area, median (IQR)	20 (15.0–25.0)
**Epilepsy Symptoms**	
Age onset of the first seizure in all PWE, median (IQR)^d^	10.0 (6.0–15.0)
Age onset of the first nodding seizure, median (IQR)	7.0 (5.0–10.0)
Onset of the first seizure last year, *n* (%)	13 (1.6)
Seizures in the last year, *n* (%)	729 (91.8)
Sudden loss of consciousness, *n* (%)	738 (92.5)
Loss of bladder control #, *n* (%)	458 (62.1)
Foaming at the mouth #, *n* (%)	654 (88.6)
Biting of the tongue #, *n* (%)	499 (67.6)
**Most Frequent Seizure Types**	
Generalized convulsive seizures, *n* (%)	614 (80.3)
Atonic seizures (drop attacks), *n* (%)	79 (9.9)
Absences, *n* (%)	79 (9.9)
Nodding seizures, *n* (%)	317 (39.7)
Focal motor seizures with decreased consciousness	4 (0.5)
Focal motor seizures without loss of consciousness, *n* (%)	1 (0.1)
**Frequency of Seizures**	
Daily seizures, *n* (%)	210 (26.8)
Weekly seizures, *n* (%)	191 (24.4)
Monthly seizures, *n* (%)	342 (43.7)
Yearly seizures	39 (5.0)
Experienced seizure in the last 12 months, *n* (%)	729 (91.8)
**Family Members with Epilepsy**	
Family history of seizures, *n* (%)	483 (60.8)
Siblings (brother/sister) *, *n* (%)	366 (75.6)
Father *, *n* (%)	8 (1.6)
Mother *, *n* (%)	1 (0.2)
Grandparent *, *n* (%)	49 (10.1)
**Seizures/Head Nodding Triggers**	
Spontaneous (no obvious trigger), *n* (%)	666 (83.7)
Sight of food, *n* (%)	134 (16.8)
Cold weather, *n* (%)	114 (14.3)
New moon appearance, *n* (%)	29 (3.6)
Sunlight/sunset, *n* (%)	10 (1.2)
**Psychomotor Development during Childhood**	
Normal growth prior to the seizure onset, *n* (%)	722 (90.8)
Normal psycho-motoric development prior to the seizure onset, *n* (%)	715 (89.9)
Intellectual disability prior to seizure onset, *n* (%)	63 (7.9)
**Severe Disease Preceding the Onset of Epileptic Seizures**	
Measles, *n* (%)	39 (4.9)
Malaria, *n* (%)	110 (14.2)
Encephalitis/meningitis, *n* (%)	19 (2.4)
Head injury with loss of consciousness, (%)	16 (2.0)
Prolonged post-traumatic coma, *n* (%)	6 (0.7)
Tuberculosis, *n* (%)	2 (0.2)
Persistent headache, *n* (%)	3 (0.2)
Diarrhoea, *n* (%)	4 (0.5)
**Physical Examination**	
Blind, *n* (%)	32 (4.0)
Facial abnormalities, *n* (%)	18 (2.2)
Cervical lymph nodes, *n* (%)	12 (1.5)
Nakalanga features, *n* (%)	51 (6.4)
Itching, *n* (%)	302 (37.8)
Burn lesions, *n* (%)	115 (14.4)
Papular/nodular pruritic skin, *n* (%)	63 (7.9)
Leopard skin, *n* (%)	15 (1.9)
Dry, thickened, or wrinkled skin, *n* (%)	30 (3.8)
Neurological Examination **	
Disoriented in place/time/person, *n* (%)	86 (10.8)
Paresis, *n* (%)	27 (4.6)
Behavioural problem, *n* (%)	51 (8.7)
**Epilepsy Classification**	
Epilepsy without head nodding, *n* (%)	446 (55.9)
Head nodding, *n* (%)	82 (10.3)
Head nodding plus other seizure types, *n* (%)	270 (33.8)
**Current Seizure Medication**	
Never used anti-seizure medication, *n* (%)	112 (14.0)
Traditional treatment, *n* (%)	1 (0.1)
Ever used anti-seizure medication, *n* (%)	15 (1.9)
Currently use anti-seizure medication, *n* (%)	644 (80.7)
**Anti-Seizure Medication**	
Phenobarbital, *n* (%)	89 (11.1)
Phenytoin, *n* (%)	32 (4.0)
Carbamazepine, *n* (%)	545 (68.3)
Sodium valproate, *n* (%)	5 (0.6)
**Ivermectin Use**	
Ever taken ivermectin, *n* (%)	661 (82.8)
Never received ivermectin, *n* (%)	95 (11.9)
**Ivermectin Intake the Year before the Survey**	
No ivermectin, *n* (%)	119 (14.9)
Ivermectin intake, *n* (%)	645 (80.8)
Ivermectin intake not known, *n* (%)	34 (4.3)
Meeting the OAE criteria, *n* (%) ***	556 (78.4)

*n*: count; IQR: Interquartile range; #:60 missing; * denominator families with seizure history (*n* = 470); ** missing neurological exam information (*n* = 214); *** missing information on OAE criteria (*n* = 89).

**Table 4:** Multivariate logistic regression model assessing risk factors for epilepsy.

Parameter	Estimate	95% CI		*p*-Value
Intercept	0.006	0.002	0.018	<0.001
Age (years)	1.291	1.194	1.397	<0.001
Age × age (years)	0.995	0.993	0.997	<0.001
Male vs. female	1.239	1.065	1.441	0.006
Farming vs. no farming	0.996	0.832	1.192	0.963
Fishing vs. no fishing	0.409	0.079	2.112	0.286
Employee vs. non-employee	1.212	0.377	3.893	0.747
Cattle keeping vs. no cattle in the households	0.744	0.297	1.863	0.528
Skin itching vs. no itching	1.308	1.102	1.552	0.002
Blindness	2.031	1.280	3.222	0.003
Recent ivermectin vs. no ivermectin intake	1.112	0.916	1.349	0.283
Distance to the river (kilometer)	0.986	0.978	0.993	0.001

CI: confidence limits; age × age: quadratic effect of age to account for non-linear relationship with age and logit of living with epilepsy.

**Table 5:** Ov16 seropositivity and ivermectin intake of children 3–9 years old without epilepsy living in neighbourhoods of Mvolo centre, close to the River Naam.

Age (Years)	Ov16 Positive, *n* (%, 95% CI)	Ivermectin Use, *n* (%, 95% CI)
3 (*n* = 26)	3 (11.5, 4.0–28.9)	
4 (*n* = 24)	5 (20.8, 9.2–40.5)	
5 (*n* = 25)	8 (32.0, 17.2–51.6)	6 (24.0, 11.5–43.4)
6 (*n* = 22)	7 (31.8, 14.3–47.6)	7 (31.8, 14.3–47.6)
7 (*n* = 15)	7 (46.7, 24.8–68.9)	7 (46.7, 24.8–68.9)
8 (*n* = 20)	4 (20.0, 8.1–41.6)	6 (30.0, 14.5–51.9)
9 (*n* = 18)	7 (38.8, 20.3–61.4)	9 (50.0, 29.0–70.9)
Overall (*n* = 150)	41 (27.3, 20.8–34.9)	

## Data Availability

The datasets generated during and/or analysed during the current study are available from the corresponding author on reasonable request.

## Supplementary Materials

The following are available online at https://www.mdpi.com/article/10.3390/pathogens10050599/s1, Supplementary Material S1: Household survey questionnaire (screening for epilepsy) Supplementary Material S2: Neurology questionnaire (stage 2).
