# Supplementary material for: High Prevalence of Epilepsy in an Onchocerciasis-Endemic Area in Mvolo County, South Sudan: A Door-To-Door Survey
Source: Pathogens. 2021 May 14;10(5):599. doi: 10.3390/pathogens10050599 (PMC8157079; doi:10.3390/pathogens10050599)
Supplement: Supplementary file 1 [file pathogens-10-00599-s001.zip › S1 HOUSEHOLD SURVEY QUESTIONNAIRE (SCREENING FOR EPILEPSY).pdf]

HOUSEHOLD SURVEY QUESTIONNAIRE: SCREENING FOR EPILEPSY

Investigator ID: ____ Signature: ____________________

Village: __________________________ Date current visit (DD/MM/YYYY): __ __/__ __/__ __ __ __

Previous HOUSEHOLD CODE __ __ __ / __ __ /__ __ __ Date previous visit __ __/__ __/__ __ __ __ GPS________________

1) Household Head : ____ Etnicity________________

2) Does family originate from this villag ☐ YES ☐ NO If NO, How long have they been residing in this village? ____________ (years)

3) Main income generating activity of the famil

Farming cattle pigs fishing shop employe soldier other, specify____________________

4) Has anybody developed died in the household since last survey?

If YES, Name : ______age ___________sex______When _____month_____year

4) Has anybody developed epilepsy/nodding in the household since last survey?

If YES, Name : ____age ___________sex______When _____month_____year

1. No. pers.		2. Age	3. Gender		4.1 QUESTION 1			4.2 QUESTION 2			4.3 QUESTION 3			4.4 QUESTION 4			4.5 QUESTION 5			4.6 Itching			4.7 Blind	4.8 IVM
Pst	Abs	Year: Y<br>Month: M	M=Male<br>F=Female		Loss of consciousness with either urine on self and/or drooling?			Absence(s) or loss of contact with the surrounding of sudden onset and brief duration?			Jerking or uncontrolled abnormal movement (convulsion) of the limb(s) of sudden onset and lasting for a few minutes?			Head nodding?			Has it ever been said that he/she is epileptic or has he already had 2 or more seizures?						1 or 2 eye	IVM in 2019?
1 ☐	☐		M ☐	F ☐	YES ☐	NO ☐	? ☐	YES ☐	NO ☐	? ☐	YES ☐	NO ☐	? ☐	YES ☐	NO ☐	? ☐	YES ☐	NO ☐	? ☐	YES ☐	NO ☐	? ☐		
2 ☐	☐		M ☐	F ☐	YES ☐	NO ☐	? ☐	YES ☐	NO ☐	? ☐	YES ☐	NO ☐	? ☐	YES ☐	NO ☐	? ☐	YES ☐	NO ☐	? ☐	YES ☐	NO ☐	? ☐		

PERSONS SUSPECTED TO HAVE EPILEPSY (REFER TO DOCTOR / NEUROLOGIST) :

CODE (I/VV/HHH/N°): __ __ __/__ __/__ __ __/__ __ Name: ______________________________ Sex (M/F): ____ Age: ________
CODE (I/VV/HHH/N°): __ __ __/__ __/__ __ __/__ __ Name: ______________________________ Sex (M/F): ____ Age: ________
CODE (I/VV/HHH/N°): __ __ __/__ __/__ __ __/__ __ Name: ______________________________ Sex (M/F): ____ Age: ________
CODE (I/VV/HHH/N°): __ __ __/__ __/__ __ __/__ __ Name: ______________________________ Sex (M/F): ____ Age: ________
