# Supplementary material for: High Prevalence of Epilepsy in an Onchocerciasis-Endemic Area in Mvolo County, South Sudan: A Door-To-Door Survey
Source: Pathogens. 2021 May 14;10(5):599. doi: 10.3390/pathogens10050599 (PMC8157079; doi:10.3390/pathogens10050599)
Supplement: Supplementary file 1 [file pathogens-10-00599-s001.zip › S2 Paper-based questionnaire for suspected epilepsy cases.pdf]

Neurology questionnaire (stage 2)

DATE : _____ / _____ / ________

PARTICIPANT IDENTIFICATION

1. Participant ID: Number AS (3 capital letters) number village (2 capital letters) number household (3 digits) number family member (2 digits)
2. First Name: ____
3. Surname Name: ____
4. Age years (if less 1year put 0)
5. If less than 1 year ……………. month
6. Gender ☐ male ☐ Female
7. Ethnic group: ____
8. Was the person living in the village since birth ☐YES ☐NO ☐DON'T KNOW
9. If not, since how long does the person live in the village
   ________ years (if less than 1year put 0)
10. What is the interviewed participant's duration of stay in the survey area __________years

HISTORY OF EPILEPSY

SEIZURE TYPOLOGY

11. Has the participant ever suddenly lost consciousness ☐YES ☐NO ☐DON'T KNOW
    If yes, did s/he experience any of the following?
    i. Loss of bladder control? ☐YES ☐NO ☐DON'T KNOW
    ii. Foam at the mouth? ☐YES ☐NO ☐DON'T KNOW
    iii. Biting of the tongue? ☐YES ☐NO ☐DON'T KNOW
12. Has the participant ever experienced absence(s) or sudden loss(es) of contact with the surroundings, for a short duration of time? ☐YES ☐NO ☐DON'T KNOW
13. Does the participant have a history of head nodding?
    ☐ YES, in the past ☐ YES, still ongoing ☐ NO ☐ DON'T KNOW
    IF YES, specify age of the participant at onset of head nodding ______ years
    If the nodding stopped, at what age did it stop? ______________years
14. Has the participant ever experienced sudden, uncontrollable twitching or shaking of arms, legs or head, for a period of a few minutes with amnesia (deficit of memory)? ☐YES ☐NO ☐DON'T KNOW
15. Does the participant sometimes experience sudden and brief bodily sensations, see or hear things that are not there, or smell strange odours? ☐YES ☐NO ☐DON'T KNOW
16. Has the participant ever been told that he / she is suffering from epilepsy or that he / she has had epileptic fits? ☐YES ☐NO ☐DON'T KNOW
    If so was the diagnosis confirmed by a medical doctor? ☐YES ☐NO ☐DON'T KNOW?
17. What type are currently the most frequent seizures (more than one answer possible)

☐ Generalized convulsive seizures
☐ Atonic seizures (drop attacks)
☐ Absences
☐ Nodding seizures
☐ Focal motoric seizures without loss of consciousness
☐ Focal motoric seizures with decreased consciousness
☐ One seizure
☐ Others, specify:______________________________
☐ No seizure. If no, skip to General examinations

18. At what age did the seizures start? ____ year ☐ DON'T KNOW (999) ☐ NA (Not applicable)(888)
19. Did the seizures start less than one year ago? ☐ YES ☐ NO ☐ DON'T KNOW
20. If yes, since how many months? __________________Months
21. What triggers the seizures / head nodding? (tick all that apply)

☐Spontaneous (no obvious trigger)
☐Sight of food ☐Cold weather
☐DON'T KNOW
☐ Other, specify________________________

SEIZURE HISTORY

22. What is the number of epileptic seizures since onset? ☐ Two ☐ Three or more seizures

If only two seizures, were they more than 24h apart? ☐ YES ☐ NO ☐ DON'T KNOW ☐ NA

23. Has the participant had a seizure in the last 5 years? ☐ YES ☐ NO ☐ DON'T KNOW
24. Has the participant had a seizure in the last 12 months?☐ YES ☐ NO ☐ DON'T KNOW
25. What is the current frequency of the seizures?

☐ Every day
☐ Every week
☐ Every month
☐ Every year

Specify number: _____________

26. a. How many seizures did you have LAST WEEK?

☐ None ☐ 1-4 episodes ☐ More than 4 ☐ DON'T KNOW

b. What is the average duration of a seizure episode?

☐ less than a minute ☐ 1-5 minutes ☐ more than 5 minutes ☐ DON'T KNOW

MEDICAL HISTORY

27. Family history of seizures ☐ YES ☐ NO ☐ DON'T KNOW

IF YES, specify who these are (tick all that apply)

☐Siblings (brother/sister); No. of affected siblings ______
☐Father ☐Mother ☐Grandparent(s)
☐ Other, Specify __________________________________

Pregnancy and Birth:

28. Did the pregnancy of the mother of the participant proceed normally? ☐ YES ☐ NO ☐ DON'T KNOW
    If NO, specify: ___________________________________
29. Was the participant born at term (pregnancy had completed 9 months)? ☐ YES ☐ NO ☐ DON'T KNOW
30. Was there a delayed cry at birth? ☐ YES ☐ NO ☐ DON'T KNOW

Psychomotor Development during Childhood:

Prior to onset of seizures

31. Was the child growing normally prior to the onset of the seizures? ☐ Yes ☐ No ☐ DON'T KNOW
    IF NO, at what age did the abnormal growing appear? ______ years
32. Did the child learn to do things like other children of his/her age prior to the onset of the seizures?
    ☐ Yes ☐ No ☐ DON'T KNOW
    IF NO, at what age did the learning difficulty start? ______ year
33. Compared with other children of his/her age, did the child appear in any way mentally backward, dull or slow before the onset of the seizures? ☐ Yes ☐ No ☐ DON'T KNOW
    IF YES, at what age did it start? ______ years

Occurrence of severe disease in the past:

34. Has the interviewed participant suffered from severe measles preceding the onset of epileptic seizures?
    ☐ YES ☐ NO ☐ DON'T KNOW
    If yes, how long before the onset of seizures ……………………Years
35. Has the interviewed participant suffered from a severe form of malaria (hospitalized) preceding the onset of epileptic seizures? ☐ YES ☐ NO ☐ DON'T KNOW
    If yes, how long before the onset of seizures ……………………Years
36. Has the interviewed participant suffered from encephalitis/meningitis preceding the onset of epileptic seizures? ☐ YES ☐ NO ☐ DON'T KNOW
    If yes, how long before the onset of seizures ……………………Years
37. Has the participant had a head injury with loss of consciousness preceding the onset of epileptic seizures?
    ☐ YES ☐ NO ☐ DON'T KNOW
    If yes, how long before the onset of seizures ……………………Years
38. Has the participant had a prolonged posttraumatic coma before the onset of epileptic seizures?
    ☐ YES ☐ NO ☐ DON'T KNOW
    If yes, how long before the onset of seizures ……………………Years
39. Was the onset of epilepsy following another illness?
    ☐ YES ☐ NO ☐ DON'T KNOW
    If YES, specify the illness: ____
40. **PHYSICAL EXAMINATION Done** ☐ YES ☐ NO if no go to (61)
41. Vision ☐ Normal ☐ Reduced ☐ BLIND, one eye affected ☐ BLIND, both eyes affected
42. Thoracic/spinal abnormalities ☐ YES ☐ NO ☐ DON'T KNOW
    IF YES, specify ____
43. Facial abnormalities ☐ YES ☐ NO
    IF YES, specify ____

44. Does the adolescent (> 16 years old) /adult looks like a child? ☐ YES ☐ NO ☐ NA
45. If yes, external signs of sexual development conform to age:
    ☐ YES ☐ NO ☐ EXAMINATION DECLINED ☐ NA
    If NO, specify:
46. girls: ☐ breast not developed ☐ NA
47. girls and boys: ☐ no pubic hear ☐ NA

48. Cervical Lymph nodes ☐ YES ☐ NO
49. Itching ☐ YES ☐ NO
50. Burn lesions ☐ YES ☐ NO

51. Dermatological examination (several answers possible) ☐ Normal ☐ papular/nodular pruritic skin ☐ leopard skin ☐ dry, thickened, wrinkled skin ☐ other skin abnormality

    Other dermatological lesion (specify) ________________________

NEUROLOGICAL EXAMINATION

52. Is the participant alert? ☐ YES ☐ NO
53. Fully oriented in place/time/person ☐ YES ☐ NO
54. Is the participant's mentally retarded? ☐ YES ☐ NO

55. Generalised muscle wasting ☐ YES ☐ NO
56. Paresis ☐ YES ☐ NO
57. Is the participant walking normally? ☐ YES ☐ NO ☐ DON'T KNOW
58. If no specify_____________________________________

Psychiatric symptoms

59. Does the participant suffer from any behavioural problem? ☐ YES ☐ NO
    IF YES, specify: ____

60. <u>Physical / Functional Indices</u>

Modified Rankin Scale: Please mark the most accurate description of the current functional state of the person with epilepsy, as observed during the evaluation

1	No significant disability despite symptoms; able to carry out all usual duties and activities
2	Slight disability; unable to carry out all previous activities, but able to look after own affairs without assistance
3	Moderate disability; requiring some help, but able to walk without assistance
4	Moderately severe disability; unable to walk without assistance and unable to attend to own bodily needs without assistance
5	Severe disability; bedridden, incontinent and requiring constant nursing care and attention

61. ***Epilepsy CASE CLASSIFICATION***
- ☐ Head nodding syndrome
- ☐ Head nodding syndrome plus (including other seizure types)
- ☐ Epilepsy without head nodding
- ☐ Other diagnosis

62. If NO epilepsy: other diagnosis?
- ☐ One seizure
- ☐ Recurrent febrile convulsions
- ☐ Dizziness / syncope
- ☐ Paroxysmal vertigo
- ☐ Alcohol/drug use
- ☐ Severe anaemia
- ☐ Severe protein malnutrition
- ☐ psychogenic non epileptic syndrome (PNES)
- ☐ Mental retardation without epilepsy
- ☐ Psychiatric illness without epilepsy
- ☐ Classic migraine
- ☐ Other, specify______________________

ANTI-EPILEPTIC TREATMENT

63. What is or was the type of seizure medication taken by the participant?
- ☐ No treatment ☐ DON'T KNOW
- ☐ Traditional ☐ anti-epileptic drug
- ☐ Mixed ☐ NA

If No treatment or Traditional or Don't know or NA: Go to Ivermectin use (66)

64. *If anti-epileptic drug treatment:* Which substance is taken by the participant (more than one answer possible)
- ☐ Phenobarbital
- ☐ Sodium valproate
- ☐ Phenytoin

☐ Carbamazepine
☐ Other anti-epileptic

If other, specify: ____

65. Is the participant taking the treatment regularly?
☐ currently (took it every day last week) ☐ only in the past ☐ DON'T KNOW

If only in the past, why?
☐ Personal reasons
☐ (Temporary) non-availability of medication
☐ Lack of financial means to buy medication
☐ DON'T KNOW
☐ Other, specify ______________________________

IVERMECTIN USE

66. Has the participant ever received ivermectin? ☐ YES ☐ NO ☐ DON'T KNOW ☐ NA

IF YES: how many years the person took ivermectin ____________years

Has the participant taken ivermectin in 2019?

☐ YES ☐ NO ☐ DON'T KNOW

Full physician name ________________________________
